# Influence Factors of the Pharmacokinetics of Herbal Resourced Compounds in Clinical Practice

**DOI:** 10.1155/2019/1983780

**Published:** 2019-03-05

**Authors:** Shi Sun, Yifang Wang, Ailing Wu, Zhen Ding, Xinguang Liu

**Affiliations:** ^1^Department of Pharmacy, Luoyang Orthopedic Hospital of Henan Province, Orthopedic Hospital of Henan Province, Luoyang, China; ^2^Institute of Integrative Medicine, Dalian Medical University, Dalian, China

## Abstract

Herbal medicines have been used to prevent and cure diseases in eastern countries for thousands of years. In recent decades, these phytotherapies are becoming more and more popular in the West. As being nature-derived is the essential attribute of herbal medicines, people believe that taking them for diseases treatment is safe enough and has no side-effects. However, the efficacy of herbal resourced compounds (HRC) depends on the multiple constituents absorbed in the body and their pharmacokinetics. Thus, many factors will influence the clinical practice of HRC, i.e., their absorption, distribution, metabolism, and excretion (ADME). Among these factors, herb-drug interaction has been widely discussed, as these compounds may share the same drug-metabolizing enzymes and drug transporters. Meanwhile there are many other potential factors that can also change the ADME of HRC, including herb pretreatment, herb-herb interactions, pathological status, gender, age of patient, and chemical and physical modification of certain ingredients. With the aim of ensuring the efficacy of HRC and minimizing their clinical risks, this review provides and discusses the influence factors and artificial improvement of the pharmacokinetics of HRC.

## 1. Introduction

The history of people employing herbal medicines can be dated back as early as 2100 B.C. in ancient Asian countries [1]. Nowadays, approximately 25% of common medications contain herbs, and this proportion has been elevated to 30% and to 50% in China, especially [2]. Not only in the East, herbal medicines have contributed the largest proportion to complementary and alternative medicine consumption in the United States and about 20% of people have taken some herbal supplementation [2, 3].

With the increasing knowledge of diseases treatment, people found that pharmacokinetics of HRC and their tissue distribution behaviors are crucial to their pharmacological efficacy [4]. For instance, differences in physiological status of body such as gender, age, diseases, and external stimulus may influence the oral bioavailability, tissue distribution, half-time (t_1/2_), maximum plasma concentration (C_max_), and time to reach C_max_ (T_max_), etc. of drugs or herbal medicines, and these changes in intrinsic pharmacokinetic parameters will cause variations in their therapeutic effects [4–8].

Meanwhile, unlike the widely employed chemical drugs, herbal medicines containing thousands of constituents are regarded as performing holistic effects through interactions among multiple active components and multiple targets [9]. Meanwhile, the internal metabolism processes of herbal medicines are complex due to these interactions, which may influence metabolism-related biological active substances, such as cytochrome P450 enzyme (CYP450) and P-glycoprotein (P-gp) [10]. Certainly, if herbal medicines were applied in combination with conventional drugs, the risk of possible interactions between constituents is increased. In addition, not all herb-drug or herb-herb interactions are harmful. Under some circumstances, these interactions can improve the bioavailability of target compounds and minimize side-effects of toxic ingredients [11, 12].

The pharmacokinetic changes of HRC are closely related to its pharmacodynamics, and the factors affecting the process* in vivo* are very complex and easy to be ignored. Thus, the aim of this review is to describe the common factors that influence the pharmacokinetics of HRC, thereby giving some references to ensure the safety and efficacy of these medications.

## 2. The Influence of Processing on the HRC Pharmacokinetics

The processing of traditional Chinese medicines (TCM) is a routine procedure and is usually performed on raw herbs before clinical use. Various traditional methods have been applied to herbs processing, such as sautéing with Chinese rice wine or brine solution, stir-heating, frying with sand, salt, honey or bile, steaming with water, ginger juice or vinegar, and sulfur fumigation [13–19]. The purpose of herb processing is modifying the nature of crude herbal materials, which results in enhancing their therapeutic effects, as well as reducing their toxicity. The content of some ingredients in herbs may increase, and others may decrease or even disappear after processing. Changing the chemical profile usually influences the pharmacokinetics of HRC.

Wine is one of the most popular processing adjuvants. Tao et al. [20] compared the pharmacokinetic differences between crude and wine-processed* Dipsacus asper *(DA) in rats. After being processed, the contents of phenolic acids of DA were decreased more than those of the crude herb, while the contents of saponins and iridoids were increased significantly. Compared to rats in the group of crude herb administration, area under the plasma concentration-time from zero to the last quantifiable time-point (AUC_0-t_) values along with C_max_ values of most compounds increased remarkably after wine-processed DA aqueous extracts administration. These differences might be attributed to the facilitating effect of wine that made ingredients absorb into the circulation more easily. Wine-processed herb exhibited more loose tissues, more small pores, larger total surface area, and smaller fractal dimension than those of crude herb, which allows the solvent to penetrate the loose tissue and to change internal structure, then increasing dissolution of herb containing components [21], and this might be another reason to explain this phenomenon. Similar results were observed in wine-processed* Rhizoma Coptidis* and* Schisandra Chinensis fructus *[22, 23].

The same adjuvant used in herbal materials processing can have different effects on pharmacokinetics of herbs. Vinegar-baked is another routine technology for herb preparation. On the one hand, during the procedure of vinegar processing, the hydrolyzation of saponins, flavonoids, and polysaccharides occurs, and the content of these components may be changed, because the main component of vinegar is acetic acid; this will result in deglycosylation of natural products with glycoside structure. The reaction makes the pharmacokinetic parameters of compounds significantly different between crude and vinegar-processed herbs and strengthens the effect of processed herbs [24]. On the other hand, some vinegar-processed herbs show less toxicity than crude herbs. This may be caused by the destruction of prototypes of some ingredients possessing the most toxicity and decreasing the bioavailability of toxic components after processing [14].

Animal-derived materials, such as pig's bile and mutton-fat, are a class of distinctive adjuvants in herb processing. There are researchers believing that bitter bile could increase the Cold nature of herbs and influence the energy metabolism of experimental animals [25]. Furthermore, a comparative pharmacokinetic study revealed that bile-processed* Rhizoma Coptidis *could increase the absorption rate of main active alkaloids into the plasma of heat syndrome rats more than raw herb [19]. These results are probably because alkaloids in* Rhizoma Coptidis *are hydrophobic, but when they meet the bile acids in processing adjuvant pig's bile, soluble salts are formed and then facilitate the water dissolving of these alkaloids, which finally leads to the absorption rate improvement of alkaloids by rats and strengthens their specific therapeutic effects.

In recent decades, a controversial processing technology named sulfur fumigation has taken the place of natural drying of postharvest medicinal herbs under sun or in the shade [26]. For one thing, this operation can make herbs look whiter and prettier and prevent them from insects and mildew with shortened drying time. For another, this processing procedure can cause chemical alteration of the herbs' origin ingredients, generate sulfonated derivatives, and then influence the pharmacokinetics of certain components [26–28].* Radix Paeoniae Alba *(the root of* Paeonia lactiflora* Pall., PA) is the most representative medicinal herb that is always processed by sulfur fumigation. Some researchers suggested that sulfur fumigation could increase the absorption time and improve the bioavailability of the active components of PA [29], whereas another study showed that the safety and efficacy of PA were reduced after this processing procedure [28]. In consideration of the debatable safety of sulfur-fumigated medicinal materials, most herbs are forbidden to be processed by sulfur fumigation in China now. Meanwhile, the permitted herbs should have sulfur dioxide residual amount less than 400 mg/kg, but this residue limitation lacks scientific evidence [26]. Overall, in order to standardize the practice of sulfur fumigation and ensure the safety and efficacy of sulfur-fumigated herbs, further studies are needed.

Different from traditional processing, new methods like ultrafine powders of Chinese herbs (D 90 < 45 *μ*m) have made great progress in clinical use for their convenience for carrying and oral administration [30]. As the pulverized herbal medicine owns a relatively larger surface area than traditional applied forms, bioavailability of many constituents* in vivo* increased [30, 31]. Consequently, this feature will help patients in taking lower dosage of herbal medicine in prescriptions and saving cost, which may improve medication compliance to ensure therapeutic effects

## 3. The Influence of Coadministration with Herbal Medicines or Drugs

In general, many people believe that herbal remedies present moderate and harmless effects to patients. Admittedly, the use of herbal medicines alone may not be dangerous, but they ignore the fact that herbal medicines contain various constituents with multiple pharmacological actions on the body. If conventional drugs are taken in combination, probable interactions of pharmacokinetics and/or pharmacodynamics may occur between them. A report reveals that nearly 80% of the world's population take herbs as their primary medications. In particular, older people tend to become the largest consuming groups of herbal prescriptions due to their commonly multiple health problems, such as chronic diseases [32]. They often take herbal medicines coupled with conventional medications, which is raising the potential for herb-drug interactions. Therefore, the risk of possible interactions between drug and co- or preadministration herbal medicine, single substance, and other components in traditional herbal compound prescriptions, even occurring in the multiconstituent herb itself, should not be disregarded. Several reviews have discussed the issue of herb-drug interactions [3, 10, 32–35]. In these reviews, they mainly focus on the effects of natural products on the pharmacokinetics or pharmacodynamics of drugs. However, on the one hand, the coadministration of chemical drug may also influence the pharmacokinetics of HRC; on the other hand, the interactions between HRC are also little discussed. Hence, we will place especial emphasis on herb-herb interactions and multicomponent interaction in an herb in this section.

### 3.1. Herb-Drug Interaction

The metabolism of HRC* in vivo* mostly depends on common drug-metabolizing enzymes and transporters, such as CYP450 or P-gp. Many papers reported that the coadministration of herbal medicines interferes in the pharmacokinetics of chemical drugs because of their sharing the same metabolizing enzymes and transporters, while the chemical drugs would also affect the pharmacokinetics of HRC by the same mechanism. Therefore, intensive studies are needed to ensure the safety and effectiveness of drugs when they coadministrated with herbal medicines.

CYP450 is a group of oxygenases, which plays a key role in the metabolism of endogenous substances and exogenous xenobiotics. Meanwhile, approximately 90% of current drugs are metabolized by CYP450 subtypes [10]. If the chemical drugs inducted or inhibited specific CYP450 isoforms, the metabolism of coadministration HRC would be influenced. Yin and Cheng et al. [36] used CYP450 probe drugs as a tool to investigate the effects of notoginsenoside R1 on the activities of several CYP450 isoforms* in vivo*. The results exhibited that compared with the pharmacokinetic parameters of the control group, the C_max_ and area under the plasma concentration-time from zero to infinity (AUC_0-*∞*_) of caffeine, which is mainly metabolized by CYP1A2, were increased, while the total plasma clearance (CL) was decreased in the notoginsenoside R1 treated group. However, other probe drugs corresponding to CYP2C11, CYP2D1, and CYP3A1/2 like tolbutamide, metoprolol, and dapsone were not affected by notoginsenoside R1 administration. These consequences indicated that patients who took drugs metabolized by CYP1A2 together with notoginsenoside R1 should evaluate the potential herb-drug interactions and be paid more attention.

Drug transporters are another important group that affects the metabolism of drugs. Until now, a lot of transporters have been characterized in humans, while P-gp is the most extensively studied one and affects the bioavailability of many oral medications [33].* Schisandra chinensis *is a commonly used herb and its extract was reported to regulate P-gp along with other transporters such as multidrug resistance-associated proteins and organic anion transporting polypeptides, as well as some drug-metabolizing enzymes. Therefore, coadministration of* Schisandra chinensis* and other drugs which are substrates of the reported transporters and enzymes may cause unfavorable herb-drug interactions [37].

Although many studies indicate that herbal medicines could alter the pharmacokinetics of coadministration drugs [38, 39], there are still some reports suggesting that no significant influence is observed in combination use of herbs with drugs [40, 41].

### 3.2. Herb-Herb Interaction

According to many fork medicine theories, the most dominant clinical application form of herbal medicines is “formula”, which is prescribed in combination of two or more herbs. Compatibility of multiple herbs is a key characteristic of formula and has exhibited its enormous influence in long-term clinical practices. It depends on both the clinical efficacy and the properties of each herb and possesses intention to obtain synergistic therapeutic effects and minimize or diminish the possible side-effects [42–44]. The chemical material basis of compatibility may be due to their interactions between the multiple constituents in the compound prescription, sequentially influencing the ADME of individual active ingredients. Hence, it is meaningful to reveal the complex mechanism of formula compatibility. Some common herb-herb interactions are summarized in Table 1.

Many works have been demonstrated to study the regularity of recipe composition. Da Chuanxiong decoction consists of* Gastrodia elata* Bl. (GE) and* Ligusticum chuanxiong* Hort. (LC), and Hu et al. compared the pharmacokinetics of gastrodin after orally administrating GE extract alone and in combination with different components of LC in rats [54]. They found that total phenolic acids and alkaloids but not tetramethylpyrazine of LC significantly affected the pharmacokinetic parameters of gastrodin. Another case investigated the pharmacokinetic compatibility of several ingredients in Sheng Mai San, a compound prescription consisting of* Panax ginseng*,* Ophiopogon japonicus*, and* Schisandra chinensis* and exhibiting curative effects on cardiovascular diseases [44]. Experiment results showed that* Schisandra* lignans extract could significantly enhance the exposure of several ginsenosides both* in vitro* and* in vivo*. Recently, a comparative pharmacokinetic study in rats was carried out to evaluate the herb-herb interactions in Guanxin Shutong Capsule (GSC) following oral administration of single herb extract and different herb extract combinations [45]. With the composing of* Choerospondias axillaris*,* Salvia miltiorrhiza Bunge*,* Syzigium aromaticum* (SA),* Borneolum syntheticum* (BS), and* Tabaschir*, GSC has been used for treating cardiovascular-related disease in clinical practice. As a result, GSC treated group showed significant promotion of the bioavailability of eugenol and reduction of the rate of its elimination processes, and the AUC_0-t_, AUC_0-*∞*_, and C_max_ of bicyclic monoterpenes (isoborneol, borneol, and camphor) were more prominently decreased than in SA-BS coextract treated group. As the accumulation of bicyclic monoterpenes proved to be toxic in long-term administration, the reduced absorption of these compounds owing to the herb-herb interactions in GSC could alleviate the toxicity of bicyclic monoterpenes to some extent. From the cases discussed above, we can speculate that improving the exposure of some bioactive components and reducing toxic ingredients absorption may be possible explanations to elucidate the mechanism of formula compatibility.

Although many researchers suggested that interactions between herbs in compound prescriptions are of common occurrence [44–47, 50–54], herbs demonstrate almost no interference with each other in some cases. Li et al. [61] reported that Honghua constituents demonstrated nearly no influences on the metabolism of Danshen polyphenols from Danhong injection via monitoring the plasma concentrations of eight Danshen polyphenols and comparing their pharmacokinetic parameters between Danshen injection and Danhong injection treated rats. Therefore, the interaction among herbs cannot be generalized, and specific discussion is required.

Nowadays, the safety of herbal medicines has been attracted more and more attention, and confirming the compatibility of herbs is a key point to ensure the safety of clinical usage of herbal remedies. “Eighteen Incompatible Medicaments” is the typical representative of TCM incompatibility. The theory proposes that specific agents in the eighteen-herb list can produce toxicity if they were used in combination. Gansui-Gancao is an incompatible herb pair recorded in “Eighteen Incompatible Medicaments”. Gansui is the root of* Euphorbia kansui* T.N. Liou ex T.P. Wang (GS) and exhibited a notable efficacy in treating malignant pleural effusion, but its efficacy could be weakened and even caused serious toxicity when used in combination with gancao, the root of* Glycyrrhiza uralensis* Fisch. or* Glycyrrhiza glabra* L (GC) [62, 63]. In general, these two herbs will not appear in one TCM formulae. Interestingly, a compound prescription called Gansuibanxia decoction (GSD) is composed of the tuber of* Pinellia ternata* (Thunb.) Breit. (BX) and the root of* Paeonia lactiflora* Pall. (SY), along with the incompatible herb pair of GS and GC, and the decoction exhibits great curative effect on phlegm retention syndrome. This is a surprising example, and it seems to violate the principles of formulating prescription. Cui et al. [48] elucidated the reasonability of this application of GSD from the pharmacokinetic prospective. They found that GS could inhibit the absorption of liquiritigenin, isoliquiritigenin, liquiritin, glycyrrhetinic acid, and glycyrrhizic acid of GC, which reduced the detoxification ability of GC and increased the toxicity of GS immediately. Nevertheless, SY demonstrated the opposite effects on the bioavailability of the main bioactive components of GC and alleviated the absorption inhibition of GS on GC in GSD. These results provided a possible explanation of this application of the incompatible herb pair.

Synergistic effect is another pivotal characteristic of TCM and demonstrates enhancement of their therapeutic effects; for example, Danshen-Sanqi (the root and rhizome of* Salvia miltiorrhiza *Bge. and the root and rhizome of* Panax notoginseng* (Burk.) F. H. Chen) and Zhishi-Baizhu (the immature fruit of* Citrus aurantium* L. or* Citrus sinensis *Osbeck and the root of* Atractylodes macrocephala *Koidz) exert synergistic actions to treat coronary heart disease and functional dyspepsia, respectively [64, 65].* Cortex Mori *(CM), the root bark of* Morus alba* L, exhibits *α*-glycosidase inhibition effect and plays an important role in regulating the postprandial blood glucose level. As the main biological active constituent of CM, 1-Deoxynojirimycin (DXM) is considered as a potent *α*-glycosidase inhibitor. Coadministration of* Radix Pueraria* (the root of* Pueraria lobata* (Wild) Ohwi, RP) flavonoids with CM extract could reduce the absorption rate of DXM significantly and thus elevate the relative concentration and duration of DMX in small intestine, which demonstrated a stronger hypoglycemic effect of CM extract compared with the herb administration alone [49]. These results agree with the principle of composition of TCM, referring to enhancing the efficacy and reducing the toxic side-effects.

### 3.3. Multicomponent Interaction in an Herb

Since multiple components constitute the connotation of herbal medicines, possible interactions between complex ingredients in a single herb may occur. Ma et al. [12] reported that interaction between stilbene glucoside and emodin, two major constituents of* Radix Polygoni Multiflori *(the root of* Polygonum multiflorum *Thunb.), was observed. This interaction subsequently elevated the degree of absorption of emodin into rat plasma and prolonged its duration time* in vivo *after the stilbene glucoside treatment. The mechanism might involve inhibition of UDP-glucuronosyl-transferases 1A8 and thus prohibit the glucuronidation of emodin. Sinomenine is the prime alkaloid of* Sinomenium acutum*, Rehder & E.H. Wilson (SA). Co-dosing sinomenine with SA extract reduced the C_max_ and AUC_0-t_ in rat plasma by comparison with pure sinomenine treated group, especially at the higher dosage of 60 mg/kg. These results suggested that the SA extract was able to decrease the bioavailability of its main constituent [60]. Similar results were observed in coadministration of pure osthole and* Libanotis buchtormensis *supercritical extract [55]. Of course, there are studies suggesting that interactions between components can also increase the absorption and improve the bioavailability of other components [11, 56, 57]. In addition, some reports indicate that the pharmacokinetic parameters of a class of compounds having similar characteristics, like flavonoids, tend to be affected together by coexisting compounds in rats [58, 59]. These results may be attributed to the competition or inhibition of the same transporters between those certain group compounds and other complex ingredients in one herb. The function and direction of interaction are uncertain, further studies should be carried out monitoring the clinical use of herbal medicines to ensure their safety and efficacy.  Table 2 represents possible interactions among multiple components in single herb.

## 4. The Influence of Pathological Status

Pharmacokinetics of certain compounds may be influenced by the pathological status of host [5, 66]. For the past few years, many researches have focused on this issue. They found that pathological factors such as liver injury, diabetes, stroke, fever, rheumatoid arthritis, migraine, coronary atherosclerotic heart disease, cancer, and neurodegenerative diseases demonstrate deep impact on metabolism of HRC [8, 67–75]. Hepatocytes are the parenchymal cells of liver which can improve the excretion of xenobiotics through urine or feces by modifying their structure, including phase I xenobiotic metabolism like oxidation or hydrolysis, followed by phase II metabolism like glucuronidation, sulfation, acetylation, or glutathione conjugation. Meanwhile, cytochrome P450 enzymes are mainly located in the pericentral area of the liver lobule, and glutathione peroxidase shows a higher expression in periportal zone [76]. So drugs are primarily metabolized in liver as this important organ generates the highest drug-metabolizing activity. Therefore, liver lesions will result in changing the pharmacokinetics of drugs [8, 77]. For instance,* dl*-Praeruptorin A (PA) is the prime active ingredient of* Peucedanum praeruptorum* Dunn and the substance of CYP 450 isozymes 3A1 and 3A2 in rats. A comparative pharmacokinetic experiment was conducted in liver cirrhosis and normal rats with single-dose intravenous administration to evaluate the pharmacokinetic variability of PA under hepatic damage condition. Compared to the control group, PA exhibited significant higher AUC_0-*∞*_ and slower hepatic elimination rate in model group. Those results might be partly caused by the lower hepatic blood flow rate and levels of CYP450 isoforms in liver cirrhosis rats [78].

Diabetes mellitus has become one of the most widespread metabolic diseases in the world, and patients suffering from type 2 diabetes mellitus (T2DM) approximately account for 90% of the diabetic totality [79]. In terms of metabolism, T2DM can induce gastrointestinal impairments, resulting in changes of the gut microbiome and slowing gastric emptying. Meanwhile, nephropathy and liver disease can be also observed in T2DM patients [80–82]. These physiological changes will affect the ADME of HRC. Wei et al. [80] invested the pharmacokinetic alteration of Sanhuang Xiexin decoction (SXD) extracts between T2DM and normal rats. They found that, compared to the control group, the AUC, C_max_, t_1/2_, and T_max_ of the six main components of SXD, namely, rhein, baicalin, wogonoside, berberine, palmatine, and coptisine, were remarkably enhanced in T2DM rats after oral administration of SXD. These results indicated that the bioavailability of the target compounds was improved and the elimination was slower in T2DM rats. Similar metabolism process* in vivo* was observed in cyanidin-3-O-glucoside and* Maydis stigma* extract treated T2DM rats [82, 83].

Stroke is one of the most serious causes of death in China and the United States [84]. Moreover, this malignant disease can cause hepatic dysfunction, affecting the secretion of glucocorticoid and gastric mucus, suppressing the gastric mucus bicarbonate barrier function and the peristalsis of stomach and small intestine. These alterations may increase bioavailability of HRC and prolong their retention time in the body [70, 85]. Meanwhile, stroke can activate a sequence of cascade reactions and damage the blood brain barrier (BBB). This makes it easier for HRC to cross BBB, thus changing the distribution of HRC [84].

It is worth noting that some model-inducement agents, such as streptozotocin and nitroglycerin, can elicit liver and kidney damage and increase microvascular permeability [80, 86]. Streptozotocin induces insulin deficiency due to its selective pancreatic *β*-cell cytotoxicity caused by DNA alkylation and nitric oxide generation. And diabetes causes structural and functional abnormalities in the liver by affecting glycogen and lipid metabolism. Nitroglycerine will induce vasodilation due to the vascular dilatory response of the brachial artery. These compounds also affect the metabolism of HRC. The metabolic changes caused by model-inducement agents do not agree with normal pathological process; perhaps some of the metabolism changes are not caused by the diseases themselves, so the results in these studies inducing models with nonself substances should be confirmed and evaluated.

## 5. The Influence of Physical and Chemical Modifications of Natural Product

Although many herbal medicines demonstrate good biological activities in tests* in vitro*, the* in vivo* assays do not exhibit reproducible results [87]. This may be attributed to the diversity of various constituents of herbal remedies, which affect the bioavailability, the internal duration, and the amount reaching the target tissue of the curative compounds. Many efforts have been made trying to solve the mentioned problems above, concerning physical and chemical modifications of active candidates derived from herbal medicines, to develop various drug delivery systems, (DDS) and change their properties and behaviors* in vivo*.

### 5.1. Promoting Bioavailability and Internal Duration of Natural Products

Most HRCs, such as flavonoids, tannins, and terpenoids, possess high water solubility or high molecular size; thus it is difficult for them to cross cell lipid membranes, leading to decreased bioavailability and efficacy [87]. Nevertheless, ingredients with hydrophobic property, like *β*-elemene, also affect their oral absorption due to the poor water solubility [88]. Low bioavailability seems to become the biggest obstacle of herbal medicines application in treating disease and brought about many problems in clinical trials [89]. Meanwhile, fast systemic clearance of HRCs also limits their therapeutic usage [90].

Poly lactic-co-glycolic acid (PLGA) is a widely used class of polymers and has been approved by US Food and Administration and European Medicine Agency for developing therapeutic nanoparticle DDS for their good biocompatible and biodegradable properties. The biodegradation process of PLGA occurs by hydrolysis and generates lactic acid and glycolic acid, which finally enter the tricarboxylic acid cycle being metabolized into carbon dioxide and water [91–93]. So, the polymers are safe enough and usually used to improve bioavailability and solubility of certain drugs [94]. Solvent evaporation, nanoprecipitation, and emulsification-diffusion technologies are commonly employed methods of PLGA nanoparticles synthesis [91]. Curcumin is a widely concerning polyphenol, which is derived from the herbal spice* Curcuma longa* L., and exhibits many physiological activities such as antioxidant, anti-inflammation, and antitumor. However, this promising bioactive compound exhibits low bioavailability and a short half-life [92]. To overcome the shortages of curcumin and improve its therapeutic effect, Tsai et al. [92] designed curcumin-loaded PLGA nanoparticles (C-NPs). The* in vivo* test results showed that the curcumin exposure (AUC/dose) dramatically increased, 55% and 21-fold, after intravenous and oral administration of C-NPs more than conventional curcumin in rats, respectively. Meanwhile, C-NPs treated rats demonstrated 22-fold relative higher oral bioavailability, extended retention time, and decreased excretion of curcumin than those of rats in the control group. All the results above revealed that C-NPs could prolong internal retention time and elevate bioavailability of curcumin. Another attempt aiming to improve the oral bioavailability of resveratrol, a poorly water-soluble anti-inflammatory and antioxidant compound, was performed via formation of resveratrol-loaded galactosylated PLGA nanoparticles (RGP-NPs) [95]. These newly synthesized RGP-NPs exhibited more than 3 times higher oral bioavailability of resveratrol than those of rats dosing resveratrol suspensions alone, as well as exhibiting increased anti-inflammatory efficacy. From these examples, we can see clearly that PLGA-conjugates significantly affect the metabolism of modified HRC.

Emulsions are a class of mixtures consisting of two immiscible liquids and stabilizing with surfactants or emulsifiers. These DDS can be divided into two types, oil-in-water (O/W) and water-in-oil (W/O), and O/W type holds dominant position in parenteral or oral administration [91, 96]. Due to the hydrophilic property and smaller particle size, emulsions can be used to deliver many hydrophobic HRCs and enhance their bioavailability and* in vivo* duration time by turning them into dissolved forms, increasing their intestinal epithelial permeability, and decreasing their hepatic uptake [97–100]. The common methods to prepare emulsions in laboratory are sonication and homogenization, with high-pressure homogenization and microfluidization on a large scale [91]. Ligustrazine is an active alkaloid derived from* Ligusticum wallichii *Franchat and has various biological effects on cardiovascular and neurovascular disorders. However, like curcumin, the low oral bioavailability and short* in vivo *half-life of ligustrazine require multiple doses to obtain optimum clinical efficacy, but this application also ascends the its toxic risk to patients [100]. Wei et al. developed a ligustrazine-loaded lipid emulsion (LLE) and invested the influence of pharmacokinetics and tissue distribution of this application form on ligustrazine. Compared with routine ligustrazine injection, the optimized LLE demonstrated a sustained release profile* in vitro*, as well as an enhanced bioavailability and improved distribution pattern in all rat tissues* in vivo*. These results made lipid emulsion a potential delivery system of ligustrazine for its clinical use [100]. Moreover, Ke et al. [99] designed a cyclovirobuxine D-loaded self-nanoemulsifying DDS and significantly enhanced the relative bioavailability of the loaded drug to 200.22% in comparison with the commercial dosage form in rabbits. Besides carrying monomers, emulsion DDS can be also loaded with multiple phytochemicals like the total flavones of* Hippophae rhamnoides *L and* Corydalis decumbens* (Thunb.) Pers. extracts, improving the relative bioavailability of those hydrophobic ingredients [98, 101]. These results show the great potential of emulsion in developing DDS for poorly water-soluble HRC.

PEGylation technique, which refers to covalent attachment of polyethylene glycol (PEG) chains to target compound with ester bonds, is a widely used chemical functionalization method of biomolecules to improve their stability, water solubility, and pharmacokinetic properties such as t_1/2_ and CL [102, 103]. Moreover, PEGylation molecules often demonstrated advantages in being protected against enzymatic degradation, as well as reducing immunogenicity and toxicity compared to their parent compounds [103, 104]. So many distinguishing properties make PEGylation technology very suitable to be applied to HRC modification. Lu et al. [102] synthesized PEGylated triacontanol (PEG-TA) as prodrug of triacontanol (TA), which exhibited antibacterial, antioxidant, antisenescence, etc. activities with low water solubility, established a gas chromatography tandem mass spectrometric method, and finally applied it to the pharmacokinetic study of PEG-TA and its metabolite TA in rats. Comparing the pharmacokinetic parameters, involving C_max_, T_max_, AUC, and mean residence time (MRT), between PEG-TA and TA treated rats via oral dosing and intravenous injection, they found that administration of PEG-TA in both ways conspicuously enhanced exposure levels and prolonged plasma half-life of TA. Namely, these results indicated that PEGylation might be a potential way to improve the pharmacodynamic properties of TA and promote its application.* Radix Ophiopogonis *polysaccharide (ROP) is a natural fraction possessing great therapeutic efficacy on myocardial ischemia. However, the poor oral bioavailability and short half-life limit its clinical application. In consideration of overcoming aforementioned shortages of ROP, Lin et al. synthesized two forms of PEGylated ROP. Finally, they found that these two newly synthesized conjugates exhibited approximate 11-13 times longer t_1/2_* in vivo* than ROP alone, showing good absorption following subcutaneous administration [105]. In addition, other technologies like micronization, salt formation, and hydroxypropyl-*β*-cyclodextrin inclusion were reported to enhance oral bioavailability of target compounds [106].

### 5.2. Target Delivering of HRC

The poor concentration of drug in target tissue prevents many drugs from exerting their therapeutic effects [107]. Hence, researchers took more and more attempts to apply plenty of methods to solve this problem. Among them, drug-targeting, including passive targeting and active targeting, is a promising technology that improves the bioavailability of HRC in desired loci* in vivo*, as well as reducing toxicity due to the localized area release of certain constituents of herbal medicines [89].

Since tissue lesions are direct manifestations of many diseases, tissue-targeting is the most important strategy in targeting DDS design. Meanwhile, the changes of tissue distribution of loaded HRC are obvious. For instance, Huang et al. [108] developed a bone-targeting liposome loaded with icaritin, an osteogenic flavonoid isolated from* Herba Epimedii*. They found that the developed targeting DDS could increase distribution of icaritin to the bone and enhance bone formation in ovariectomized mice compared to the control group. In addition, other cases also indicated tissue-targeting DDS can enhance the therapeutic effects of loaded HRC on certain disease and, of course, proved changes of their tissue distribution indirectly [109–111].

Cells are the basic structures and functional units of organisms. Interfering with the physiological activities of pathological cells has become one of the means to treat diseases, especially in cancer therapy. Therefore, many cell-targeting DDS are developed to improve the bioactive HRC uptake into concerning cells. These attempts will change the distribution of loaded components between normal and diseased cells. Recognized as an anti-inflammation and anticancer agent, celastrol, an active constituent of* Tripterygium wilfordii* Hook. F., possesses the property of poor water solubility and target selectivity [112]. To surmount these challenges, Niemelä et al. designed sugar-decorated mesoporous silica nanoparticles (SDMSN) as vectors of celastrol and investigated the target-specific efficacy on induction of apoptosis of cancer cells. Consequently, the uptake of SDMSN in HeLa and A549 cancer cells was four and five times higher than mouse embryonic fibroblasts and shows no toxicity to normal cells [112]. As tumor cells extensively produce acidic metabolites and export acid to the extracellular space, these characteristics result in a peripheral acidic microenvironment around tumor cells [113]. Based on this feature of tumor cells, researchers have developed pH-sensitive DDS, and the loaded HRC release is facilitated in the acidic microenvironment of tumor, rather than normal cells, thereby changing the distribution of curative compounds and enhancing their target-specificity [114].

Organelles are fundamental structures of cells and keep cells working normally. Among them, mitochondria are very important organelles and their dysfunctions are linked with cancer, diabetes, and other diseases [115]. More and more attention has been attracted by mitochondria-mediated apoptosis of tumor cells, and researchers believe that this may be a promising approach in cancer therapy. Therefore, mitochondria-targeting DDS emerge at the right moment. Recently, glycyrrhetinic acid [116] and hypericin [117] functionalized graphene oxide carriers were reported to exhibit mitochondria-targeting property, improve mitochondrial permeability, and enhance the uptake of loaded drug into mitochondria.

## 6. Influence of Other Factors

Gender is a very important influence factor in drug metabolism, especially for women. The inter-gender pharmacokinetic alterations are mainly attributed to the differences of sex hormones secretion, the variety of intestinal and hepatic CYPs and transporters, the body fat percentage and the average body weight, plasma volume, and organ blood flow between male and female [118, 119]. These discrepancies have overall effects on drug ADME. Some reports indicate that women suffered high risk of adverse drug reaction in certain circumstances than men [118, 120]. Therefore, it is particularly important to study the sex-based impact on drug metabolism. Yang et al. [6] performed a study to investigate the gender-related differences of pharmacokinetics of diosbulbin B (DB) in rats. As a result, the female rats exhibited approximately 7 times higher oral absolute bioavailability of DB than male rats. Moreover, a bigger apparent volume of distribution (Vd), longer internal retention, and faster clearance were observed in female group after intravenous administration of DB. Xu et al. [9] explored the sex-related pharmacokinetic differences of* Schisandra* lignans after oral-dose of* Schisandra chinensis* extract in rats and found that female rats demonstrated a higher internal amount and slower elimination rate of focused compounds than male group. In detail, the t_1/2_ of all the five marker ingredients, namely, schisandrin, schisandrol B, deoxyschisandrin, *γ*-schisandrin, and schisantherin A, was 2-9 times longer, along with 5-50 times higher C_max_ and AUC_0-t_ of the tested compounds except schisantherin A, compared to those achieved in male rats. Nevertheless, it has been pointed out that gender difference did not significantly influence the pharmacokinetic parameters of paeoniflorin [121]. As the results showed above, the gender-related changes of pharmacokinetics seem to be unpredictable, so more attention is needed to explore the differences of herbal medicine applications between genders.

Age appears to be another influence factor of pharmacokinetics, for the age-related differences in gastric emptying rate, the concentration of serum proteins, and the activity levels of drug-metabolizing enzymes, as well as the function of liver and kidney [122, 123]. Specifically, children exhibit increased CL of certain drugs, while elders show the opposite trend. Meanwhile, elder people show decreased absorption rate and increased unbound drug concentration in plasma due to the slower gastric emptying and lower concentration of serum proteins than adults [122]. A population pharmacokinetic study suggested that the population estimate of the Vd of artesunate and dihydroartemisinin, two derivatives of artemisinin treating severe malaria, was higher in adults than children, but CL was not significantly changed [124]. Another population pharmacokinetic study concerning daikenchuto, a traditional Kampo used in Japan to treat various gastrointestinal complications, also revealed that age is an important index in drug metabolism [125]. In summary, dosage adjusting to different age is a vital issue during the drug treatment, and age-related pharmacokinetic changes should be taken into account.

Acupuncture is a traditional therapy and has been used in China for thousands of years. It is always applied by inserting thin needles into specific points, which is called “acupoints”, of the body of people being treated and then rolling the needles manually or simulating by electricity [126, 127]. Due to the oddity of its theory and operation method, it was considered to be a Chinese equivalent of voodoo decades ago [128]. Nevertheless, acupuncture is becoming a distinguished alternative therapy and being adopted in countries worldwide to treat chronic pain, osteoarthritis, asthma, rhinitis, heroin addiction, rheumatoid arthritis, etc. [127–129]. Moreover, acupuncture is the most popular alternative treatment in US fertility clinics for couples desiring fertility care [130]. Additionally, evidence shows that acupuncture can promote the release of several neuropeptides in the central nervous system and demonstrate meaningful physiological effects [131]. In a word, acupuncture shows great potential therapeutic effect on certain body disorders. As acupuncture is an external stimulus, it can disturb the internal balance of the body and affect the metabolism of herbal medicines. Zhou et al. [132] reported that acupuncture could improve the absorption and reduce the elimination of baicalin in normal rats, and they found that stimulating specific acupoints, such as Jizhong (Du6), Dazhui (Du14), and Zhongwan (Ren12), was able to cause a bimodal phenomenon of the concentration-time course of baicalin. The synergistic effect on pharmacokinetics was also observed in combination with acupuncture at Zusanli (ST36) and oral administration of* Schisandra chinensis* in rats, as well as improving the target tissue distribution of three main lignans of* Schisandra chinensis*, namely, schisandrin, deoxyschisandrin, and schisandrin B, in comparison with the herb-alone treated group [4, 127]. All the published data above suggests that acupuncture seems to be able to decrease the required dosage of herbal medicines, thus economizing the total amount of herb consumption and reducing the possibility of adverse drug reactions.

## 7. Conclusion

According to evidence presented in this review, numerous factors like preliminary treatment, combination with drugs or herbs, pathological status, chemical or physical modifications, age, gender, and acupuncture will influence the pharmacokinetics of herbal medicines. In particular, as aging society is coming, the population of elder people with multiple health disorders and taking multiple medications is growing larger and larger. The occurrence of interactions between herbal medicines and drugs or internal body environment should be paid more attention particularly. Knowledge of factors affecting the pharmacokinetics of herbal medicines can lead to better guidance of their rational administration, whereas studies of these factors are mainly limited to animals at present and clinical research is lacking. Therefore, clinical research is required to focus in the future on elucidating and verifying the mechanism of the interactions between the influence factors and herbal medicines. The better knowledge of factors affecting the pharmacokinetics of herbal medicines we gain, the better guidance of their rational administration we can apply.

## Figures and Tables

**Table 1:** Possible herb-herb interactions.

Herb 1	Herb 2	Monitoring indexes	Pharmacokinetic parameters of indexes comparing to administration herb 1 alone										Ref.
			AUC_0-t_	AUC_0-^*∞*^_	t_1/2_	T_max_	C_max_	MRT_0-^*∞*^_	MRT_0-t_	K	V_d_	CL	
*Borneolum*	Guanxin Shutong Capsule	Camphor	↓		↓		↓						
		Isoborneol	↓	↓	↑		↓						
		Borneol	↓	↓			↓						
		Eugenol	↑	↑	↑	↑	↓						
	*Syzigium aromaticum*	Camphor			↓		↓						[45]
		Isoborneol					↓						
		Borneol			↓								
		Eugenol				↑	↓						

*Salviae miltiorrhizae*	Borneol	Rosmarinic acid		↑			↑	↑					
		Salvianolic acid A		↑			↑						[46]
		Salvianolic acid B		↑	↑			↑					

*Radix et Rhizoma Rhei*	Dahuang-Fuzi Decoction	Chrysophanol	↓	↓	↑	↑	↓						[43]
		Physcion	↓	↓		↑	↓						

*Paeonia lactiflora*	Zengmian Yiliu Formula	Albiflorin	↓	↓			↓			↓^a^	↑	↑	[47]
		Paeoniflorin			-^c^					↓^b^			

*Radix Kansui* and *Radix et Rhizoma Glycyrrhizae*	Gansui-Banxia Decoction	Liquiritigenin	↑	↑	↑	↓	↑						
		Isoliquiritigenin	↑	↑	↑	↑	↑						
		Liquiritin	↓	↓	↑	↓	↑						[48]
		Glycyrrhetinic acid	↑	↑		↓	↑						
		Glycyrrhizic acid	↑	↑	↑								

*Radix Paeoniae*	*Radix et Rhizoma Glycyrrhizae*	Liquiritigenin	↑	↑			↑						
		Isoliquiritigenin	↑	↑			↑						
		Liquiritin	↑	↑	↑	↑	↑						[48]
		Glycyrrhetinic acid	↑	↑	↑		↑						
		Glycyrrhizic acid		↑	↑	↑	↑						

*Cortex Mori*	*Radix Pueraria* Flavonoids	1-deoxynojirimycin				↑	↑	↑					[49]

*Ramulus Cinnamomi*	*Ephedrae Herba*	Coumarin	↑	↑	↑			↑	↑				
		Cinnamic alcohol	↑	↑					↑				[50]
		Cinnamic acid	↑	↑	↓			↑	↑				

Huo Luo Xiao Ling Dan	*Radix Paeoniae Rubra*	Paeoniflorin	↓	↓		↓						↑	
		Albiflorin	↓	↓		↓						↑	
		Oxypaeoniflorin	↓	↓		↓						↑	
	*Corydalis yanhusuo*	Tetrahydropalmatine	↑	↑	↓	↓	↑					↓	[51]
		Corydaline	↑	↑		↓	↑						
		Dehydrocorydaline	↑	↑		↓	↑					↓	
		Berberine	↑	↑		↓	↑						

Huo Luo Xiao Ling Dan	*Radix Angelica sinensis* and *Rhizome Ligusticum chuanxiong*	Senkyunolide A	↑				↑						
		Ligustilide	↑	↑	↓		↑						
		Butylidenephthalide	↑	↑	↓	↓	↑						[42]
		3-butyl-phthalide	↑	↑			↑						
		Levistilide A	↑	↑	↓	↓	↑						

*Panax Ginseng* glycosides	*Schisandra lignans*	Ginsenoside Rb2^d^	↑	↑									
		Ginsenoside Rc^d^	↑	↑									
		Ginsenoside Rd^d^	↑	↑									
		Ginsenoside Rb1^e^	↑	↑									[44]
		Ginsenoside Rb2^e^	↑	↑									
		Ginsenoside Rd^e^		↑									

Quercetin and Rutin	Leaves of *Bacopa monnieri*, Fruits of *Hippophae rhamnoides *and Bulbs of *Dioscorea bulbifera*	Quercetin	↓				↓						[52]
		Rutin	↑	↑			↑	↑					

*Radix Paeoniae Alba* and* Radix et Rhizoma Glycyrrhizae *(1:1)	*Radix Paeoniae Alba* and* Radix et Rhizoma Glycyrrhizae *(4:1)	Albiflorin	↑				↑					↓	
		Oxypaeoniflorin	↑				↑					↓	
		Paeoniflorin	↑				↑					↓	
		Isoliquiritin	↑										
		Liquiritigenin	↑									↓	[53]
		Isoliquiritigenin	↓				↓					↑	
		Ononin	↑									↓	
		Glycyrrhizin	↑				↑					↓	
		Glycyrrhetinic acid	↓				↓						

*Rhizoma Tianma*	Ferulic acid	Gastrodin		↑	↑			↓					[54]
	Total phenolic acid of *Rhizoma Chuanxiong*	Gastrodin		↑			↑						

*Rhizoma Tianma*	Total alkaloids of *Rhizoma Chuanxiong*	Gastrodin		↑									[54]
	*Rhizoma Chuanxiong*	Gastrodin		↑	↑		↓	↑				↓	

Blanks indicate not mentioned or not significant changes.

a. decrease in elimination rate constant.

b. decrease in distribution/absorption rate constant.

c: decrease in elimination half-life and increase in distribution half-life.

d: single dose.

e: multiple doses.

**Table 2:** Possible multicomponent interaction in an herb.

HRC1	HRC2/Herb	Monitoring indexes	Pharmacokinetic parameters of indexes compared to administration HRC1 alone									Ref.
			AUC_0-t_	AUC_0-^*∞*^_	t_1/2_	T_max_	C_max_	MRT_0-^*∞*^_	MRT_0-t_	V_d_	CL	
Osthole	*Libanotis buchtormensis*	Osthole	↓		↑	↓	↓				↑	[55]

Epimedin C	*Herba Epimedii*	Epimedin C	↓			↓	↓			↑	↑	[56]

Tectoridin	*Iris tectorum*	Tectoridin	↑	↑	↑		↑				↑	[57]

Emodin	Stilbene glucoside of *Radix Polygoni Multiflori*	Emodin	↑	↑	↑		↑		↑	↑	↓	[12]
		Emodin glucuronide	↓	↓	↑		↓		↑	↑		

Magnoflorine	*Cortex Phellodendri*	Magnoflorine	↑	↑	↑	↑		↑	↑		↓	[11]
	Berberine	Magnoflorine	↑	↑			↑				↓	

Flavonoid fraction of *Abelmoschus manihot*	Macromolecular fraction of *Abelmoschus manihot*	Rutin				↑						
		Isoquercitrin			↓	↑	↑					
		Hibifolin				↑						
		Quercetin-3'-O-glucose				↑						
		Quercetin			↓	↑						
	Small molecule fraction of *Abelmoschus manihot*	Rutin	↑		↑		↑					[58]
		Hyperiode	↑									
		Isoquercitrin	↑									
		Hibifolin	↑			↑						
		Myricetin	↑			↑	↑					
		Quercetin-3'-O-glucose	↑				↑					
		Quercetin			↓							

	Macromolecular and Small molecule fraction of *Abelmoschus manihot*	Rutin	↑									
		Hyperiode	↑		↑	↑						
		Isoquercitrin	↑			↑						
		Hibifolin				↑						[58]
		Myricetin				↑						
		Quercetin-3'-O-glucose	↑			↑						
		Quercetin	↑		↓							

*Ginkgo* flavonoids	*Ginkgo biloba*	Apigenin	↑			↓	↑		↓	↓	↓	
		Luteolin	↑			↑	↑		↑	↓	↓	
		Naringenin	↑			↑			↓		↓	
		Quercetin			↑	↑	↑		↑	↑	↓	[59]
		Diosmetin	↑			↑	↑			↓	↓	
		Kaempferol	↑				↑		↑		↓	
		Isorhamnetin	↑				↑			↓	↓	

Sinomenine	*Sinomenium acutum*	Sinomenine	↓	↓			↓			↑	↑	[60]
